# Factors influencing public support for dairy tie stall housing in the U.S.

**DOI:** 10.1371/journal.pone.0216544

**Published:** 2019-05-07

**Authors:** Jesse A. Robbins, Caitlin Roberts, Daniel M. Weary, Becca Franks, Marina A. G. von Keyserlingk

**Affiliations:** Animal Welfare Program, Faculty of Land and Food Systems, University of British Columbia, Vancouver, British Columbia, Canada; University of Illinois, UNITED STATES

## Abstract

A number of studies have shown widespread public concern over housing animals in ways that restrict their ability to move freely. Dairy cows housed in tie stall barns are tethered continuously or for part of the day, but no study has assessed public support for this type of housing system. We report two experiments assessing public perceptions of tie stall housing for dairy cattle using a hypothetical referenda format. In Experiment 1, 65% of participants (*n* = 430) said they would support a ban on tie stalls. The probability of supporting a ban increased as the duration of time that cows were tethered increased. In Experiment 2, information about possible economic consequences was included. Relatively fewer (55%) participants (*n* = 372) indicated they would support a ban. Supporters of a ban were willing to pay an average dairy product price premium of 68% to see the ban enacted. Indirect measures of support indicated socially desirable responding was greater in Experiment 2 where the economic impacts of voting behavior were made explicit. In both studies, women and liberals were more likely to support a ban. The majority of participants in Experiment 1 (51%) and Experiment 2 (57%) said they had never heard or read anything about tie stalls before participating in our survey. We conclude that current knowledge of the use of tie stalls is low, but if this situation were to change there may be considerable public concern about the use of this housing method.

## 1.0 Introduction

Restrictive housing systems have been the primary focus of high-profile animal welfare campaigns targeting food animal industries. In the US, ballot initiatives have resulted in 33 separate state animal welfare initiatives; the majority of which have focused on restrictive housing systems used by the swine and egg laying industries [1]. The emphasis on restrictive housing systems seems to reflect widespread public sentiment that animals lacking the opportunity to move freely and express normal, social behaviors have reduced levels of welfare [2]. In one recent survey of US citizens the ability of animals to interact socially was perceived as the most important factor for ensuring the welfare of dairy cattle [3]. Collectively there is a growing body of evidence that restrictive housing is a source of public controversy.

Tie stalls are the primary form of housing on 39% and 75% of US and Canadian dairy farms, respectively [4,5]. Unlike other common housing systems (e.g. free stalls, dry lots, compost barns, etc.), cows in tie stalls are restrained or tied in their individual stalls. Like most housing systems, tie stalls have advantages and disadvantages in terms of animal welfare. Compared to cows housed in free stalls, tie stall-housed cows tend to have lower levels of lameness [6]. However, comparing housing systems is complicated because farms using tie stalls differ in other respects that may be protective against lameness. For example, cows housed in tie stalls are also more likely to have access to exercise areas or pasture–which has been shown to reduce lameness [7]. Another study, reported tethered cows were motivated to move and thwarting this ability likely leads to frustration. However, they did not find any effect of tethering on acute or chronic physiological stress response measures [8].

Although tie stall housing has received little attention in North America, relative to other forms of restrictive farm animal housing (e.g. gestation stalls and battery cages; [9]), it has been a topic of controversy in other parts of the world. Tie stall housing was the predominant housing type in many European countries (e.g. 75% of all Swedish dairy herds [10] and 88% of Norwegian dairy cattle [11]), until a decade ago when their use declined (see [12]) as the result of moratoriums on the construction of new tie stall barns based partly on animal welfare concerns (e.g. [13,14]).

Unlike restrictive housing systems common to other species, some farms using tie stalls allow cows time outside of the stall for some part of the day (e.g. 73.1% of tie stall operations in the US reported having allowed pasture access for their lactating herd; [4]), a practice that may make tie stalls more acceptable to the public [15]. Moreover, expensive modifications can also reduce public support insofar as they result in increased costs for animal products [16].

To our knowledge, there has not yet been any research examining public perceptions of tie stall housing for dairy cattle in the US, let alone how this support may vary depending on how much outdoor access the cows are provided. In the two experiments reported here we assess support for tie stall housing by asking participants how they would vote if presented with the ballot initiative to ban this housing. Our primary aim was to assess public support for tie stalls, and how the duration of tethering affects this support. Our secondary aim was to determine if information about price increases resulting from a ban would affect how participants voted. We hypothesized that support for a ban on tie stall housing would increase with the time cows were described as being tethered, and that support would decrease with increased costs associated with implementing this ban.

## 2.0 Materials and methods

This research was approved by the University of British Columbia’s Behavioural Research Ethics Board (H17-02641). All participants provided written informed consent to participate.

### 2.1 Experiment 1

Participants for this study were recruited using Amazon’s Mechanical Turk crowdsourcing (M-turk) service. M-turk samples have been used in a variety of studies addressing farm animal welfare issues [17–19]. They have also been shown to be more diverse [20–22] and attentive [23] than traditional subject pools. Participation was limited to US residents with an M-turk acceptance rate of at least 95%. M-turk acceptance rates refers to the percentage of tasks (e.g. surveys) a subject has taken and received payment for satisfactory participation. Duplicate IP addresses were blocked from participating. To minimize potential selection bias prospective participants were simply asked if they would be willing to, “share their opinions” and thus were not aware of the specific focus of the study until after they agreed to participate.

We selected a hypothetical referenda format as our methodology to explore these questions. This approach has been employed in other research addressing animal welfare issues due to its ecological validity. Moreover, this approach focuses on behavioral intentions (e.g. voting behavior), which better predict actual behavior than attitudes [24].

To gauge issue awareness the survey instrument began by asking participants to indicate how much they had read or thought about tie stall housing for dairy cattle using a 5-point scale (1 = *None at all*, 5 = *A great deal*). Next, participants read a brief introduction informing them that their subsequent responses “may help guide dairy industry policies and practices.” Emphasizing consequentiality in such a manner has also been shown to increase the validity of hypothetical valuation experiments [25]. Since we anticipated a low level of issue awareness, all participants read a brief description of tie stall housing before answering any further questions. The description was developed with help of dairy scientists and industry representatives with diverse views about the impacts of tie stall housing on cattle welfare. Our overarching goal was to ensure the description was intelligible, accurate and reasonably balanced.

Since we could find no published information about the average duration of time cows are typically tethered in tie stalls each day, this variable was manipulated by randomly varying its value between 1 and 24 hours. After reading the description participants were directed to the next page where they were asked how they would vote if there was a referendum banning tie stalls. The order in which the dichotomous response options (e.g. “I would vote IN FAVOR”) appeared was randomized to control for a possible primacy effect [26]. The complete description and response options thus read as follows (information in ***bold italics*** indicates content that was manipulated):

Approximately 40% of US dairy farms keep their cows in what are known as "tie stall" barns. In tie stall barns, a chain or rope is attached to a collar around the cow’s neck to prevent the cow from turning around or leaving her stall. Food and water are provided at the front of the stall. On average, cows living in tie stall barns are tied for ***X*** hour(s) each day. In the remaining time cows are able to use outdoor areas. Some people feel tie stalls are unacceptable because they restrict the cow’s ability to move freely. Others feel tie stalls make it easier to monitor cows and attend to their individual needs.

Would you vote in favor or against a referendum that banned the use of tie stalls?

a) I would vote IN FAVOR of a referendum that banned the use of tie stalls

b) I would vote AGAINST a referendum that banned the use of tie stalls

To assess any social-desirability bias in responses, participants were asked to predict the proportion of their fellow survey respondents that would vote in favor of the ban using a slider scale with visible values ranging from 1–100%. Variations on this indirect measure have been applied in previous studies [27, 28]. Socially-desirable responding occurs when people attempt to align their responses with perceived social norms, rather than how they themselves actually feel. Responses to questions that ask them to forecast how others are likely to respond are thought to more closely reflect their true beliefs because they impersonal judgments lack ego-defensive and impression management incentives [29]. To encourage more effortful consideration participants were told that all correct predictions (+/- 2%) would earn a bonus of $4.00.

The survey concluded by gathering socio-demographic information on: age, gender, income, living environment (rural vs. urban), education, children, political ideology, pet ownership and dairy consumption. All participants received $0.70 USD upon completion of the survey.

### 2.2 Experiment 2

Participants in Experiment 2 followed the identical protocol described for Experiment 1, with several exceptions. Most importantly, a sentence was added that mentioned a possible dairy product price increases resulting from the ban. Following previous authors [30], the value of this price premium (PRICE) was randomly varied between 1% and 100%. The duration of time that cows were described being tied was also held constant at 10 to 12 hours. This range was selected because it approximates the interval between morning and evening milkings when cows are most likely to be released from their stalls, if released at all. The complete description thus read as follows (information in ***bold italics*** indicates content that was manipulated):

Approximately 40% of US dairy farms keep their cows in what are known as "tie stall" barns. In tie stall barns, a chain or rope is attached to a collar around the cow’s neck to prevent the cow from turning around or leaving her stall. Food and water are provided at the front of the stall. On average, cows living in tie stall barns are tied for 10–12 hours each day. In the remaining time cows are able to use outdoor areas. Some people feel tie stalls are unacceptable because they restrict the cow’s ability to move freely. Others feel tie stalls make it easier to monitor cows and attend to their individual needs.

Would you vote in favor or against a referendum that banned on use of tie stalls if it resulted in a ***Y%*** increase in the price of dairy products?

a) I would vote *IN FAVOR* of a referendum that banned the use of tie stalls

b) I would vote *AGAINST* a referendum that banned the use of tie stalls

Before making their selection, participants read a brief ‘cheap talk’ script adapted from Olynk and Ortega [31] reminding them that willingness to pay is often overstated and by agreeing to pay additional costs they will necessarily have less money to purchase other things:

“Experience from previous surveys finds people often vote differently in a hypothetical referendum (such as this one) where they don’t have to pay money, than they do in a real referendum where they actually could have to pay money as a result of their vote. It is important that you make your selection like you would if you were really going to face the consequences of your vote—which is to pay more money if the proposition passes."

Inclusion of cheap talk scripts has been shown to lead to responses that more closely reflect actual consumer behavior [32].

### 2.3 Statistical analysis

Binary logistic regression models were fitted to test the effects of tethering duration (Experiment 1) and price premium (Experiment 2) on the probability of supporting a ban on tie stalls while controlling for key socio-demographic variables (recoded for ease interpretation as described in Table 1).

**Table 1:** Explanatory variable coding used to determine factors that would influence the support for a ban on dairy tie stall housing when presented to US participants (Experiment 1: n = 430) (Experiment 2: n = 372).

Variable Name	Description/Coding
AGE	Age of respondent/Continuous(years)
FEMALE	Gender/Dichotomous (1 = Female; 0 = Male)
INCOME	Annual household income/Continuous (thousands)
KIDS	Parents/Dichotomous (1 = Yes; 0 = No)
COLLEGE	Educational attainment/Dichotomous (1 = College or more advanced degree; 0 = No college degree)
RURAL	Area lived most of life/Dichotomous (1 = Rural; 0 = urban or suburban)
LIBERAL	Political ideology/Ordinal (1 = Extremely conservative to 7 = Extremely liberal)
DAIRY	Weekly dairy consumption/Continuous (average number of times per week dairy is consumed)
PETS	Pet owner/Dichotomous (1 = Yes; 0 = No)

## 3.0 Results

### 3.1 Sample description

Standard demographic characteristics for both experiments (Table 2) compared favorably to US Census data. The median age of participants (36 years) was slightly less than the US average of 38. The sample was slightly male biased (i.e. fewer females than expected from the US average of 51%). Median household income was less (50,000 vs 55,000), and percentage of households with at least one child household was similar (40% vs 41%) to US averages. Almost half of participants (48%) had earned at least a Bachelor’s degree, which is higher than the US population average (31%). The regional distribution of participants was representative of the general US population where 17% live in the Northeast; 21% in the Midwest; 24% live in West and 38% living in the South [33]. The proportion of participants who reported living in a rural area (24%) was slightly greater than the national average (19%) [34]. Participants were more liberal than the general US population (50% vs 26%) [35]. The vast majority of participants (97%) reported consuming dairy products with 43% reporting they consumed them more than 7 times a week. The proportion of participants owning pets (68%) was identical to large scale survey data drawn from a probability sample [36]. Across both studies over half of all participants (54%) said they had not read or thought about tie stalls prior to taking the survey while only 2% said they had read or thought a great deal about them.

**Table 2:** Participant demographics for the two experiments designed to test factors affecting support for a ban on tie stall housing.

	Exp. 1	Exp. 2	Total
Characteristic	(*n* = 430)	(*n* = 372)	(*n* = 802)
Age (median years)	35.6	36.2	35.9
Female (%)	47.0	50.0	48.5
Income (median $)	48,500	50,000	50,000
Children (mean number)	0.7	0.7	0.7
Education (%)			
<High school	0.7	1.1	0.9
High school	36.5	34.4	35.5
Associate’s or trade degree	13.5	17.7	15.5
Bachelor’s degree	38.4	37.9	38.2
Graduate or advanced degree	10.9	8.9	10.0
Region (%)			
Northeast	17.5	18.4	18.0
Midwest	20.2	19.3	19.7
South	34.7	40.0	37.5
West	27.7	22.3	24.8
Living environment (%)			
Rural	23.3	24.7	23.9
Suburban	48.1	49.5	48.8
Urban	28.6	25.8	27.3
Political ideology (%)*			
Liberal	51.6	48.7	50.2
Conservative	26.5	30.4	28.3
Centrist	21.9	21.0	21.4
Dairy consumption (servings/week)			
None	3.0	3.2	3.1
1–3	21.2	19.1	20.2
4–6	31.9	27.4	29.8
7–9	23.5	26.9	25.1
10 or more	20.5	23.4	21.8
Pet owner (%)	65.3	72.0	68.3
Awareness (%)**			
None at all	51.2	56.5	53.6
A little	29.8	26.3	28.2
A moderate amount	10.9	9.1	10.1
A lot	6.7	5.3	6.1
A great deal	1.4	2.7	2.0

*Response options recoded for purposes of comparison: 1–3 = conservative, 4 = centrist and 5–7 = liberal

** Q: “How much have you read or thought about the practice of tethering dairy cows in tie stalls?”

### 3.2 Experiment 1

**Fig 1:**
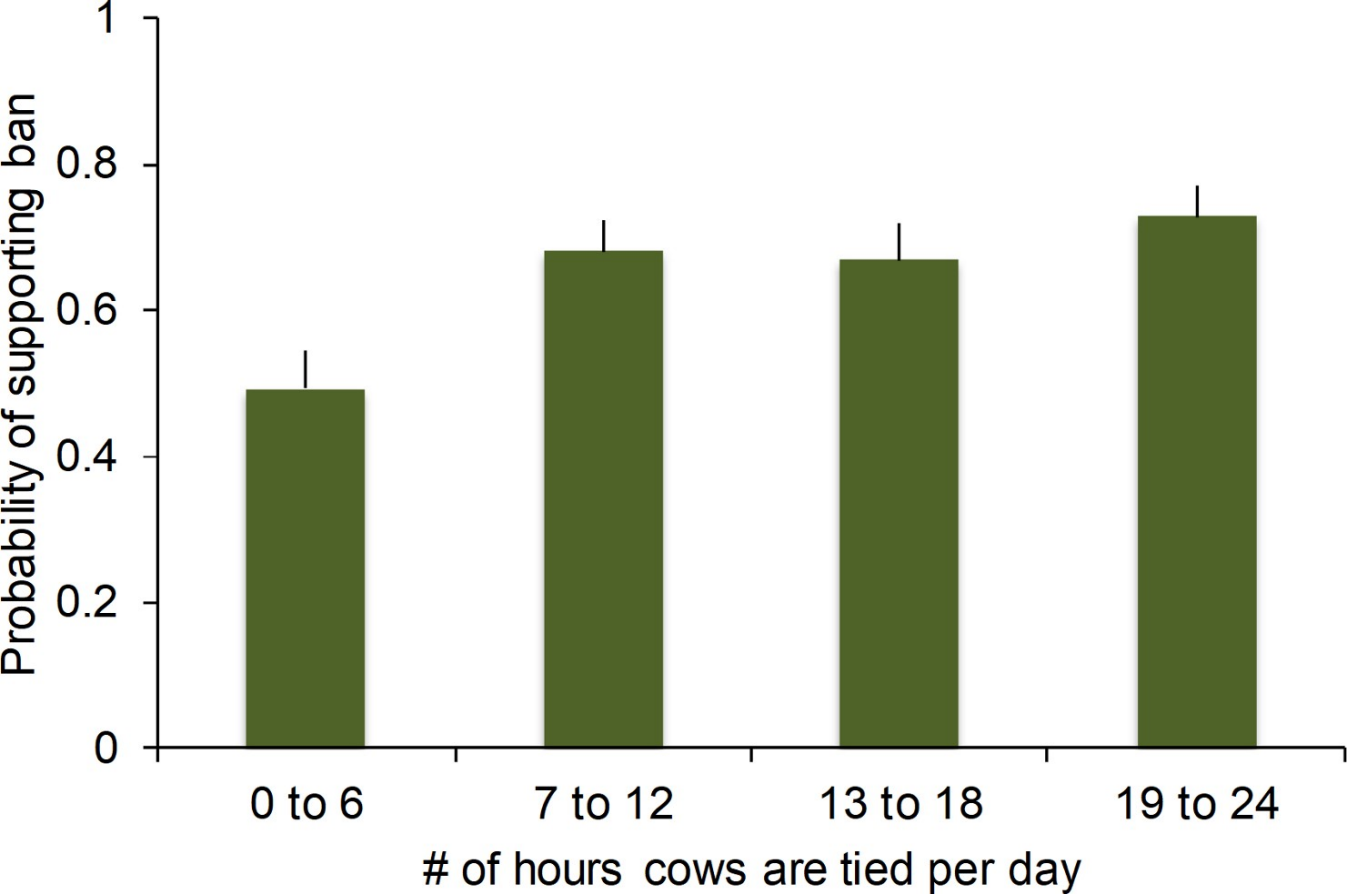
Experiment 1: the probability (mean ± SE) that participants (*n* = 430) would support a ban on the use of tie-stall housing for dairy cattle relative to the number of hours per day that the animals are tied. The number of hours that cows were tethered is illustrated on the x-axis using 6-h bins to simplify presentation, but all statistical models treated time as a continuous effect.

Participants required on average 4.4 minutes (*SD* = 3.3) to complete the survey. A total of 65% of participants indicated they would support a ban on tie stalls. Participants predicted 63% of their fellow survey respondents would support the ban. As expected, participant support increased with the number of hours cows were tethered (Fig 1, *p* < 0.01). Liberal political orientation increased the probability of supporting the ban, and female participants tended to be more supportive of the ban (Table 3); although the regression model was significant it only explained about 6% of the variation (Table 3).

**Table 3:** Experiment 1: results of logistic regression analysis (*n* = 430) testing how hours tethered and a variety of demographic factor relate to support for a ban on tie stall use. The slope (*β*), the SE of the slope, and the associated P-value are provided for each factor included in the model. Rows in **bold** (P = <0.10) indicate factors associated with support for a ban. The R^2^ for the full model was 0.06.

Variables	*β*	SE	*P*
**HOURS**	**0.04**	**0.02**	**0.006**
AGE	0.00	0.00	0.752
INCOME	-0.00	0.00	0.470
**FEMALE**	**0.37**	**0.21**	**0.085**
KIDS	-0.05	0.22	0.817
COLLEGE	-0.31	0.34	0.352
RURAL	-0.07	0.25	0.784
**LIBERAL**	**0.15**	**0.06**	**0.020**
DAIRY	-0.03	0.08	0.649
PETS	0.08	0.22	0.717

### 3.3 Experiment 2

**Fig 2:**
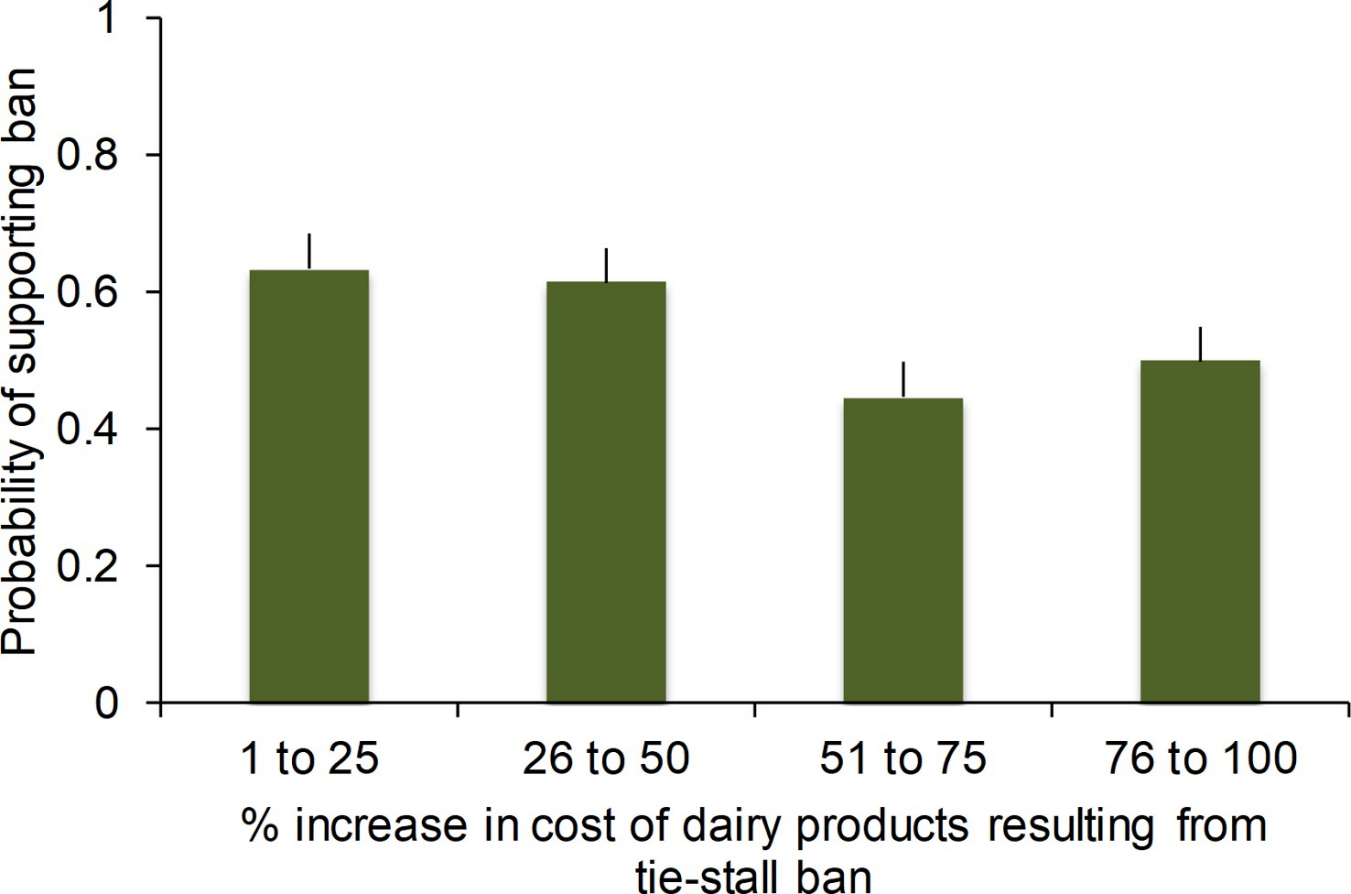
Experiment 2: the probability (mean ± SE) that participants (n = 372) would support a ban on the use of tie-stall housing for dairy cattle relative to the expected percentage increase in the price of dairy products resulting from a ban. Price increases are illustrated on the x-axis using bins of 25% to simplify presentation, but all statistical models treated price increase as a continuous effect.

Participants required on average 5.4 minutes (*SD* = 7.3) to complete the survey. In total, 55% of participants indicated they would support a ban on tie stalls. Support for a ban decreased to 44% when participants were asked to predict how others would vote. Participants were less likely to support a ban when this resulted in a larger expected increase in the cost of dairy products (Fig 2), with similar demographic effects to those described for Experiment 1 (Table 4). The full model explained 11% of the variation (Table 4).

**Table 4:** Experiment 2: results of logistic regression analysis (*n* = 372) testing how the expected percentage increase in the price of dairy products resulting from a ban, and a variety of demographic factors, relate to support for a ban on tie stall use. The slope (*β*), the SE of the slope, and the associated P-value are provided for each factor included in the model. Rows in **bold** (P = <0.10) indicate factors associated with support for a ban. The R^2^ for the full model was 0.11.

Variables	*β*	SE	*P*
**PRICE**	**-0.01**	**0.00**	**0.004**
AGE	-0.01	0.00	0.283
INCOME	-0.00	0.00	0.366
**FEMALE**	**0.69**	**0.23**	**0.002**
KIDS	0.04	0.24	0.871
COLLEGE	-0.36	0.34	0.288
RURAL	-0.37	0.26	0.152
**LIBERAL**	**0.18**	**0.07**	**0.008**
DAIRY	-0.06	0.08	0.439
PETS	-0.18	0.25	0.485

## 4.0 Discussion

Previous work has shown that many US citizens (63%) are concerned about the welfare of dairy cattle [37]. Moreover a number of studies have examined public support for specific dairy management practices such as tail docking [3,38], cow-calf separation [39,40], dehorning [3,41] and provision of pasture access [3,42]. Most of these studies have found a substantial gap between public support and current industry practice. Substantial mismatches between industry practices and societal expectations can result in diminished public trust and increased support for regulatory intervention [17].

The two studies reported here are the first to address a common dairy housing system. Our results suggest many US citizens would support a ban on the use of tie stall housing. The support for this ban is consistent with earlier work showing support for a ban on gestation stall housing for pigs [43]. The latter study showed that support decreased when participants were provided information about possible increases in the price of pork; a finding consistent with the results of Experiment 2. Taken together, these findings indicate that support for ballot initiatives declines when these are thought to result in price increases. These results also illustrate how subtle differences in framing can affect responses to surveys.

It should be noted that there is little evidence that a switch from tie stalls to less restrictive forms of housing would actually increase the price of dairy products. Indeed, very few larger farms use tie stalls, and the less restrictive systems facilitate the use technologies such as milking parlors and robotic milking machines that likely reduce the cost of production [44]. We modeled increased prices here to assess the generality of our results when this does result in a price increase. A more likely result of a ban on tie stall housing is that some (generally smaller farms) would go out of business rather than invest in new housing; future work may wish to consider how respondents would respond if this was disclosed as part of the scenario.

Results of Experiment 1 showed that participant support for a proposed ban on tie stalls (65%) was similar to participant predictions of support (63%), suggesting minimal social-desirability bias. It is worth noting that these figures are similar (63.5%) to the results of the most well-known U.S. farm animal welfare reform referendum—California’s Proposition 2—which restricted intensive housing of veal calves, egg-laying hens and gestating sows. However, in Experiment 2 self-stated support for the ban was more than 10% greater than predictions about the how their peers would respond. Thus, it appears providing participants with information about possible price increases not only decreases self-stated support for a ban, but also increases socially-desirable responding whereby participants perceive themselves as more willing to pay for associated price premiums than their peers. Epley and Dunning [45] have argued that such discrepancies are due to overly optimistic beliefs about one’s own behavior, rather than overly pessimistic beliefs about the behavior of others.

In Experiment 1 there was a trend that women were more likely to support a ban on tie stalls; this is consistent with a large body of research showing women exhibit more positive attitudes towards animals [46–49]. Recent work suggests the effect of gender is mediated by differences in empathy [50]. The relationship between gender and willingness to pay is consistent with previous research showing that women are willing to pay more for higher levels of animal welfare [51,52]. This effect of gender may be especially important given that women make the majority of household food purchasing decisions [53] and vote at higher rates than men [54].

The association between political ideology and support for a ban is also consistent with previous research showing concern for farm animal welfare is positively associated with liberal ideology [55–58]. In the context of the current work, which was posed as a referendum, it is not possible to determine the extent to which this association might be driven by underlying political differences in concern for animals or well-established differences in attitudes regarding the role of government intervention. It is also possible that a third variable correlated with both politics and concern for animal welfare such as religiosity may explain these results.

Overall awareness of the practice of tie stall housing was low. Across both experiments, when participants were asked how much they had heard or read about tie-stall housing for dairy cows, most said they had never read or heard anything at all and only 2% said they had read or heard a great deal. Given that only a small proportion of the US population is directly involved in food animal production it is not surprising that many were unaware of this specific (albeit long standing) housing method. Given the very low-level of awareness of tie-stall housing most responses were likely heavily based on the written description we provided. As with all such studies, provision of alternative descriptions, and even the inclusion of images, could alter responses. Two previous studies examined the effect of images in addition to written descriptions of pregnant sow housing systems (Canada; [59]; Brazil; [60]). In both cases the more the public learnt about the gestation stall housing system the less willing they were to accept their use. When addressing attitudes towards specific farming practices attempts should also be made to assess issue awareness so it is possible to distinguish those who have more considered opinions from those who are hearing about the issue for the first time [61].

Lastly, it should be noted that in both studies the regression models, despite being significant, only explained 6% and 11% of the variation in Experiment 1 and 2, respectively. The fact that only a small amount of the variation was explained by the demographic factors studied indicates that there may be other important factors such as attitudes towards livestock farming that are worthy of investigating.

## 5.0 Conclusion

Many US participants were willing to support a hypothetical ban on tie stall housing for dairy cattle. Support for a ban increased when number of hours that cows were tethered, and decreased when participants were told that the ban may result in increased prices for dairy products. Many participants were unfamiliar with the tie stalls before taking the survey, but these results indicate that there would be considerable opposition should the public become more aware of this housing system.

## Supporting information

S1 File Robbins et al Factors Influencing Public Support for Dairy Tie Stall Housing in the US data EXPERIMENT 1 LaTeX Source File (TEX file). (CSV)

S2 File Robbins et al Factors Influencing Public Support for Dairy Tie Stall Housing in the US data EXPERIMENT 2 LaTeX Source File (TEX file). (CSV)

S3 File Robbins et al Factors Influencing Public Support for Dairy Tie Stall Housing in the U.S. R code. (PDF)
