# Supplementary material for: Factors influencing public support for dairy tie stall housing in the U.S
Source: PLoS One. 2019 May 7;14(5):e0216544. doi: 10.1371/journal.pone.0216544 (PMC6504086; doi:10.1371/journal.pone.0216544)
Supplement: S3 File — (PDF) [file pone.0216544.s003.pdf]

R-code used for statistical analysis:

```
###Experiment 1 (ts_hours)
### table of sample descriptive stats.
install.packages("psych",dependencies=TRUE)
library(psych)
describe(ts_hours)
############################################
####convert and construct new variables###
############################################
ts_hours$female <- factor(ts_hours$female)

###make sure conversion was successful####
is.factor(ts_hours$female)

###continue of for each factor....
ts_hours$female <- factor(ts_hours$female)
ts_hours$region <- factor(ts_hours$region)
ts_hours$pet <- factor(ts_hours$pet)
ts_hours$opinion <- factor(ts_hours$opinion)
ts_hours$hrs_yes <- factor(ts_hours$hrs_yes)

###a few variables need a little extra work
ts_hours$ed <- factor(ts_hours$ed)
levels(ts_hours$ed) <- c("0", "0", "1", "1", "1", "1")

ts_hours$rural <- factor(ts_hours$rural)
levels(ts_hours$rural) <- c("1", "0", "0")
ts_hours$children <- factor(ts_hours$children)
levels(ts_hours$children) <- c("0", "1", "1", "1", "1")
```

```
ts_hours$consumption <- factor(ts_hours$consumption)

#### here: None = 0, 1-3xwk = 0, 4-6xwk = 0, 7-9xwk = 0, 10 or morexwk = 1
levels(ts_hours$consumption) <- c("0", "0", "0", "0", "1", "1", "1")

###create politics var solely for descriptive demo reporting
ts_hours$politics <- factor(ts_hours$liberal)
levels(ts_hours$politics) <-c("1", "1", "1", "2", "3", "3", "3")

###double check conversions were successful
str(ts_hours)

###Model for Experiment 1
mylogit1 <- glm(hrs_yes ~ hours + age + female + rural +
                children + income + ed + liberal + pet + consumption,
              data = ts_hours, family = "binomial")

###get R2
install.packages("BaylorEdPsych")
library(BaylorEdPsych)
PseudoR2(mylogit1)

###Experiment 2 (ts_wtp)

#############################################
####convert and construct new variables###
#############################################
ts_wtp$female <- factor(ts_wtp$female)
ts_wtp$region <- factor(ts_wtp$region)
ts_wtp$rural <- factor(ts_wtp$rural)
```

```
ts_wtp$pet <- factor(ts_wtp$pet)
ts_wtp$opinion <- factor(ts_wtp$opinion)
ts_wtp$wtp_yes <- factor(ts_wtp$wtp_yes)

###a few variables need a little xtra work
ts_wtp$ed <- factor(ts_wtp$ed)
levels(ts_wtp$ed) <- c("0", "0", "1", "1", "1", "1")
ts_wtp$rural <- factor(ts_wtp$rural)
levels(ts_wtp$rural) <- c("1", "0", "0")
ts_wtp$children <- factor(ts_wtp$children)
levels(ts_wtp$children) <- c("0", "1", "1", "1", "1")
ts_wtp$consumption <- factor(ts_wtp$consumption)
levels(ts_wtp$consumption) <- c("0", "0", "0", "0", "1", "1", "1")

###create politics var solely for descriptive demo reporting
ts_wtp$politics <- factor(ts_wtp$liberal)
levels(ts_wtp$politics) <-c("1", "1", "1", "2", "3", "3", "3")

###Model for Experiment 2
mylogit2 <- glm(wtp_yes ~ dollars + age + female + rural +
         children + income + ed + liberal + pet + consumption,
        data = ts_wtp, family = "binomial")

###get R2
install.packages("BaylorEdPsych")
library(BaylorEdPsych)
PseudoR2(mylogit2)
```
